# Molecular Mechanisms Involved in Oxidative Stress-Associated Liver Injury Induced by Chinese Herbal Medicine: An Experimental Evidence-Based Literature Review and Network Pharmacology Study

**DOI:** 10.3390/ijms19092745

**Published:** 2018-09-13

**Authors:** Cheng Zhang, Ning Wang, Yu Xu, Hor-Yue Tan, Sha Li, Yibin Feng

**Affiliations:** School of Chinese Medicine, The University of Hong Kong, 10 Sassoon Road, Pokfulam, Hong Kong, China; zttc@connect.hku.hk (C.Z.); ckwang@hku.hk (N.W.); u3004614@connect.hku.hk (Y.X.); hyhtan@hku.hk (H.-Y.T.); u3003781@connect.hku.hk (S.L.)

**Keywords:** hepatotoxicity, oxidative stress, traditional Chinese medicine, network pharmacology

## Abstract

Oxidative stress, defined as a disequilibrium between pro-oxidants and antioxidants, can result in histopathological lesions with a broad spectrum, ranging from asymptomatic hepatitis to hepatocellular carcinoma in an orchestrated manner. Although cells are equipped with sophisticated strategies to maintain the redox biology under normal conditions, the abundance of redox-sensitive xenobiotics, such as medicinal ingredients originated from herbs or animals, can dramatically invoke oxidative stress. Growing evidence has documented that the hepatotoxicity can be triggered by traditional Chinese medicine (TCM) during treating various diseases. Meanwhile, TCM-dependent hepatic disorder represents a strong correlation with oxidative stress, especially the persistent accumulation of intracellular reactive oxygen species. Of note, since TCM-derived compounds with their modulated targets are greatly diversified among themselves, it is complicated to elaborate the potential pathological mechanism. In this regard, data mining approaches, including network pharmacology and bioinformatics enrichment analysis have been utilized to scientifically disclose the underlying pathogenesis. Herein, top 10 principal TCM-modulated targets for oxidative hepatotoxicity including superoxide dismutases (SOD), malondialdehyde (MDA), glutathione (GSH), reactive oxygen species (ROS), glutathione peroxidase (GPx), Bax, caspase-3, Bcl-2, nuclear factor (erythroid-derived 2)-like 2 (Nrf2), and nitric oxide (NO) have been identified. Furthermore, hepatic metabolic dysregulation may be the predominant pathological mechanism involved in TCM-induced hepatotoxic impairment.

## 1. Introduction

It appears in early evolution that oxidative stress commonly associates with either long-term degenerative diseases or acute ischemia–reperfusion injury [1,2,3,4,5,6]. The causes for these pathologies are generally inseparable from the shift of reactive oxygen species (ROS) from mediating normal physiological responses (i.e., redox biology) to invoke inevitable cellular dysfunctions through oxidative damage, especially inefficient oxidative phosphorylation in the mitochondria [7]. ROS, characterized as a byproduct of aerobic metabolism, primarily comprises superoxide anion, hydrogen peroxide, and hydroxyl radicals that confer reactivity to numerous targets in physiological and pathological processes. Despite the fact that mammals possess well-equipped antioxidant systems, including enzymes and non-enzyme antioxidants, it remains inadequate to normalize the severe hepatotoxicity induced by toxic substances with strong pro-oxidant properties.

Treatment strategies of traditional Chinese medicine (TCM) in alleviating different diseases, such as tumor proliferation, diabetic retinopathy, and liver dysfunctions, have had prolonged utilization and are generally considered as multitarget therapies with minimal adverse actions [8,9,10,11]. In contrast to the therapeutic effects, side effects of TCM, such as hepatotoxicity, have rarely been reported. However, accumulating evidence regarding TCM-associated oxidative hepatotoxicity was frequently addressed in recent years, particularly in paradoxical pharmacological activities of toxic and curative actions [12]. The most vital clue is the association between the initiation of TCM administration and hepatotoxicity generation and, of equal importance, to the deceleration following withdrawal. Concordantly, liver is the primary organ susceptible to pathological cascades of oxidative stress. In particular, parenchymal cells are most vulnerable in an oxidative environment. Abundant ROS are produced from mitochondria, and microsomes in parenchymal cells by regulating PPARα-associated signaling pathways. In addition, in Kupffer cells, the hepatic oxygen sensor and resident liver macrophage, has been postulated to trigger the formation of hepatic fibrosis by excessive ROS-induced apoptosis and inflammation [13]. Both hepatic stellate and endothelial cells are all specialized in producing ROS in physiological and pathological systems, and accustomed to suffering from lipid peroxidation [14]. Of note, although studies in the evaluation of mammal’s susceptibility to TCM-induced oxidative hepatotoxicity have been extensively demonstrated, specific identification of potential targets in relation to pathogenesis of TCM-dependent hepatotoxicity is limited. Hereby, we performed a literature review with network pharmacology, aiming to systematically decipher the highly pathogenic molecular targets for oxidative hepatotoxicity regulated by TCM.

## 2. Molecular Mechanisms Involved in TCM-Induced Oxidative Hepatotoxicity

### 2.1. Redox Status in Physiology and Pathology

Redox signaling is of essential importance to aerobic metabolism in regulating cell functions, including signal transduction pathways, defense in response to invading microorganisms, and gene expressions for cell physiological activity [15]. Oxidative stress is one of the key pathogenic processes that mainly associates to the disorder of redox homeostasis. A high concentration of redox signaling of ROS is commonly observed with cell damage and metabolic dysregulation, including lipid peroxidation, and irretrievable protein and DNA degeneration [16]. Moreover, the trigger of oxidative stress in a coordinated manner can disseminate the impairment to extrahepatic organs, including the failure of kidney, brain, and lung, which seem to indiscriminately oxidize almost all molecules in tissues [17,18,19]. However, under normal oxygen metabolism, ROS is perceived to be a molecular secondary messenger involved in the signal transduction mechanism in response to cytokines, hormones, and adenosine triphosphate (ATP), regulating the biological and physiological processes [20]. However, excessive ROS can be efficiently scavenged through intracellular redox homeostasis to maintain the cell metabolism and survival. The antioxidant system in our body is sensitive to the alterations in redox state for alleviating potential chain reactions of oxidative stress. Therefore, whether ROS is linked to orchestrated biological processes in routine metabolism or initiation of oxidative stress depends on the steady or imbalance of redox state. Aside from the beneficial effects, disruption of redox homeostasis, by chemicals in general, is correlated to the moderate-to-severe damage in organisms which, in turn, accelerates the progression of oxidative stress-related impairment [21].

To keep the generation of ROS controllable in liver, both enzymatic and non-enzymatic systems are in charge of maintaining the redox homeostasis. The steady-state cellular redox status can malfunction when exposed to pro-oxidant xenobiotics with toxic levels [22]. More specifically, redundant ROS is generally eliminated by a series of the following enzymes, primarily containing superoxide dismutase (SOD), catalase (CAT), and glutathione peroxidase (GPx), whereas non-enzymatic molecules include glutathione (GSH), tocopherol, and beta-carotene. When the antioxidant system is vulnerable, the expression of activated ROS will be enhanced [23]. Ultimately, disruption of redox homeostasis is established with the undesirable elevated pro-oxidants. Subsequently, the physiological functions of several amino acids, such as cysteine, tyrosine, and tryptophan, are impaired. ROS-mediated pathological alterations in these amino acids are greatly susceptible to proteolytic attack via proteasomes in oxidized proteins, since proteins are composed of numerous amino acids and comparatively more prone to have specific targets of ROS [24]. An excess of ROS enhances mitochondrial permeability and functions. Reactive aldehydes, like 4-hydroxynonenal (4-HNE), can be released by ROS that inactivate the mitochondrial respiratory chain by hindering electron stream and activating oxidative stress [25].

In addition, pathological changes in sensitive proteins caused by oxidative stress can be a reversible or irreversible process. Reversible modifications are commonly implicated with cysteine for preventing the ROS-induced irreversible loss of cell function, including the carbonylation of lysine and arginine, Di-tyrosine generation, and protein–protein crosslinks, that exacerbate the degeneration and accumulation of proteins in cytoplasmic inclusions [26]. Therefore, regarding the prevention of oxidative stress in physiological circumstances, interference with superfluous pro-oxidant factors should be controlled through redox homeostasis system, which is typically composed of enzyme and non-enzymatic antioxidants.

### 2.2. Enzymatic and Non-Enzymatic System in Redox Homeostasis 

**Figure 1:**
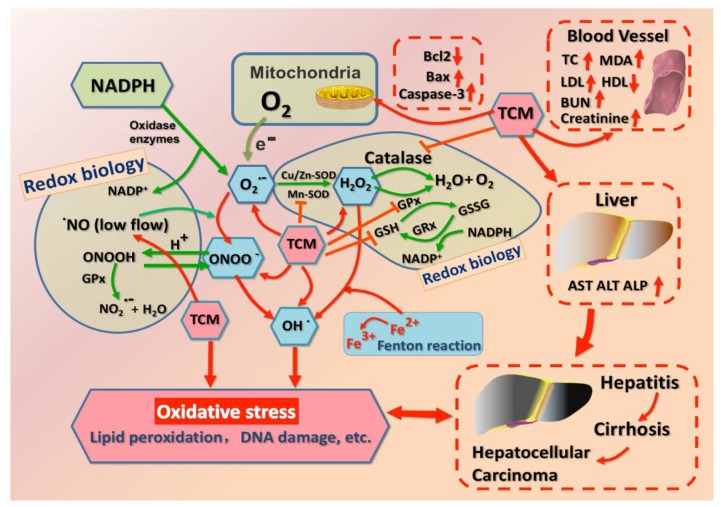
Schematic mechanisms of redox biology and traditional Chinese medicine (TCM)-induced oxidative stress in hepatocytes.

The human body possesses sophisticated mechanisms to optimally maintain the redox homeostasis and cell functions against oxidative stress. This is achieved by the generation of antioxidative substances derived from endogenous antioxidant system or producing exogenous components of enzymatic and non-enzymatic antioxidants. Enzymatic reactions mediated by antioxidant endogenous enzymes ameliorate cellular oxidative stress-induced cell death principally through scavenging toxic substances, including overproduced intracellular ROS and reactive nitrogen species (RNS), aiming to stabilize the cellular contents of DNA and protein. As shown in Figure 1, the principal antioxidant enzymes concerning the neutralization of ROS are SOD, CAT, GPx, glutathione reductase (GR), and GRx. More specifically, SOD catalyzes the dismutation of superoxide peroxide (O^2−^) to oxygen (O^2^) and hydrogen peroxide (H_2_O_2_). H_2_O_2_ is decomposed into H_2_O and O_2_ by the action of CAT and GPx. In this process, CAT is an enzyme that can be found in almost all aerobiotic organs [27], whereas GPx enzymes transform H_2_O_2_ by using it to oxidize reduced GSH into oxidized glutathione (GSSG). GR, a flavoprotein (FAD-containing) enzyme, regenerates GSH from GSSG with the reducing power of nicotinamide adenine dinucleotide phosphate oxidase (NAD(P)H) [28,29].

Notably, accumulating superoxide is the vital substance that tends to be more correlated with oxidative stress, rather than redox signaling, in the oxygen consumption of this enzymatic system. Specifically, superoxide normally consists of the one-electron reduction of intracellular oxygen that can be converted by SOD into H_2_O_2_. SOD is the main defense substance against superoxide, including two isoforms as SOD1 and SOD2. SOD1 is a homodimeric protein termed Cu-ZnSOD, and mainly distributed in the cytosol as well as mitochondrial intermembrane space. SOD1 comprises the ions of copper and zinc in cytoplasm and nucleus [30]. Copper is necessary for catalytic reaction, whereas zinc is imperative for maintaining protein structure. However, SOD-2 is defined as manganese-associated SOD (MnSOD) and primarily exists in the mitochondrial matrix. Both two isoforms possess an identical mechanism of dismutation of O^2−^ into H_2_O_2_.

Non-enzymatic antioxidants capable of rapidly inactivating free radicals and oxidants, can be categorized into two classifications, as follows: endogenous (metabolism) and exogenous (nutrient) antioxidants, in which metabolisms comprise glutathione, lipoic acid, and L-arginine, etc. In terms of their location, these proteins and molecules exert intracellular or extracellular therapeutic mechanisms to counteract excessive ROS and RNS. In addition, compounds that cannot be directly synthesized in vivo but in vitro, such as vitamin C, vitamin D, and trace metals, are identified as exogenous antioxidants [31]. As for vitamin C (ascorbic acid), it is a water-soluble, potent intra- and extracellular antioxidant that eliminates physiological free radicals, including hydroxyl and peroxyl radicals [32]. Moreover, GSH is an affluent thiol-based antioxidant inside cells, rich in live aerobic cells, and involved in both enzymatic and non-enzymatic reactions. As a cofactor for GPx, GSH catalyzes the reduction of H_2_O_2_ to H_2_O and O_2_. Furthermore, GSH can restrain the formation of the highly toxic substance hydroxyl radical through attenuating genomic instability and preventing lipids, proteins, and DNA from being indiscriminately oxidized [33,34].

### 2.3. Hepatotoxicity Caused by Specific Pro-Oxidant TCMs

Regarding the indispensable role of liver in the biotransformation of foods and medicines, hepatic disorders commonly result from the imbalance of metabolic homeostasis [35]. Although the ingestion of toxic substances, heterologous compounds, anticancer drugs, and immunosuppressive agents are known as the potential inducers of liver injury, growing evidence illustrates that long-term intake of curative drugs, such as certain anti-inflammatory and anticancer TCMs, may as well cause a large spectrum of hepatotoxicity including acute liver injury, steatosis-hepatitis, and fibrosis, etc. [36,37,38,39,40]. Furthermore, heightened reports of TCM-induced oxidative hepatic damage were particularly emphasized in recent studies [41]. In parallel, liver is the main organ of escalating ROS attack [42]. Although ROS may act in either positive or negative role on cellular functions in terms of the intensity and duration of oxidative stress, ROS production with an intoxicating dosage has been frequently detected by certain TCM treatments. Hepatotoxic TCM can irretrievably alter the biological functions of proteins, DNA contents, lipids, carbohydrates, membranes, which leads to oxidative stress-triggered hepatocyte injury [43]. However, the explicit mechanism by which overloaded ROS causes hepatic injury upon TCM treatment is not fully disclosed. Thus, searching effective approaches for identifying potential TCM with oxidative hepatotoxicity and underlying mechanisms are urgently demanded.

### 2.4. Literature Search Methodology

To further achieve an in-depth understanding of TCM-induced oxidative hepatotoxicity, series of the terms, including “Chinese medicine” or “Chinese herb” in combination with “hepatotoxicity” or “liver injury”, are firstly utilized to search the database of PubMed, Google Scholar, and Web of Science. After the initial exploration, in-text references related to these screening conditions will be selected manually. The keyword of “oxidative stress” will be further taken in the screening filter. Finally, literature that matched with all aforementioned criteria are accepted, otherwise, the articles were considered irrelevant and excluded. In addition, studies within the past 5 years are incorporated into the construction of both Table 1 and network pharmacology, which provide the up-to-date comprehension of the role of TCM-dependent oxidative hepatotoxicity. Several hepatotoxic TCMs with intensive elucidation are discussed in the following sections.

### 2.5. Pure Compounds

Tetrandrine, a principal alkaloid isolated from *Stephania tetrandra*, has revealed multiple therapeutic effects on rheumatism, glaucoma, myocardial infarction, and tumor treatments [44,45,46,47]. However, it is noteworthy that tetrandrine-related liver toxicity has been reported, and overexpression of ROS and disorder of mitochondrial permeability transition (MPT) was found to associate with tetrandrine-induced liver toxicity. MPT is a vital pathogenetic mechanism of drug-induced liver failure, and was identified by the overloaded intramitochondrial Ca^2+^-induced progressive permeabilization of the inner mitochondrial membrane, resulting in mitochondrial swelling and membrane rupture [48]. Besides, GSH depletion, as well as the activation of the pro-oxidant enzyme cytochrome P450 (CYP450) and especially cytochrome P2E1 (CYP2E1), was observed in tetrandrine-treated hepatocytes. Of note, chronic administration of tetrandrine for more than 3 months, with the dose ranging from 2 to 5 mg/kg, sensitized hepatocytes to oxidative damage [44].

Isoline and retrorsine are the pyrrolizidine alkaloid derived from the Chinese medicine *Ligularia duciformis* [49]. In spite of the curative effect of anti-inflammation and blocking cough reflex, pyrrolizidine alkaloid is commonly believed to be a representative poisonous alkaloid that disturbs the metabolism of numerous organs, especially in the liver [50]. Potential intoxication of enhanced serum alanine transaminase (ALT) and aspartate aminotransferase (AST) can be detected in clinical application as an antitussive agent. Furthermore, increased expression of GPx-1 and GST-Pi can be recognized in isoline-treated mouse liver, indicating that the self-defense to counteract oxidative stress in the body is activated, because both GPx-1 and GST-Pi are GSH-associated antioxidant enzymes. Concomitantly, upregulated malondialdehyde (MDA) and ROS, in combination with the degradation of glycine N-methyltransferase (maintaining contents of cellular folate), GPx, and CAT can be observed as well, alterations which further confirmed the soline-induced oxidative stress in hepatotoxicity [51]. Severe hepatic GSH depletion can take place as a result of the excessive production of dehydro-retrorsine generated from P450-modulated metabolic activation of retrorsine with the toxic dose of 0.2 mmol/kg, along with the abundant serum AST and ALT. Meanwhile, as covalent binding of reactive metabolites plays a vital role in the mechanism of toxic actions, higher rates of pyrrole–protein adduction than the vehicle group were observed in retrorsine-treated mouse, indicating that additional convincing evidence of *Ligularia duciformis*-caused hepatotoxicity has been provided [52,53,54].

Geniposide, an iridoid glycoside in *Gardenia jasminoides*, exerts anti-fibrotic, anti-osteoarthritis, and anti-epilepsy actions by modulation of the expression of transforming growth factor-β (TGF-β)/Smad4, p38 mitogen-activated protein kinase (p38 MAPK), and PI3K/Akt/GSK-3beta signaling pathways, respectively [55,56,57]. Whereas, geniposide manifested a number of pathological phenomena in the liver of SD rat with the dose greater than 574 mg/kg, such as elevated MDA, liver enzymes (ALT, AST, alkaline phosphatase (ALP), total bilirubin), focal necrosis, and downregulated SOD, leading to the onset of oxidative stress caused hepatotoxicity [58]. Meanwhile, exposure to geniposide less than 574 mg/kg was considered non-toxic to the liver, according to the unaltered serum biochemical indicators and liver weight [58].

Saikosaponins, an oleanane-type triterpenoid saponins, is the major bioactive ingredient extracted from *Radix bupleuri*, which has been used to prevent Alzheimer’s, pulmonary diseases, and even viral hepatitis [59,60,61]. However, in accordance with the cumulative evidence, saikosaponins probably contributed to toxicity in hepatocytes, and in particular, caused acute liver injury with doses of more than 19 g/day for a human being with 70 kg body weight. Metabolic dysregulation of lipids and proteins can be taken place due to excess ROS generation in the treatment of saikosaponins. Saikosaponins dose- and time-dependently evoked the increase of AST, ALT, and lactate dehydrogenase (LDH) [62]. CYP2E1, an important member of cytochrome P450 mixed function oxidase enzymes, plays a critical role in the metabolism of xenobiotics. Abundant ROS was detected, along with the upregulating CYP2E1 that linked to the dysregulated lipid metabolism upon saikosaponins treatment. Saikosaponin-induced oxidative stress was further proved by the dose-dependent depletion of GSH and elevation of MDA and inducible nitric oxide synthase (iNOS) [62].

Vincristine, a major compound of *Catharanthus roseus*, has been demonstrated to potently attenuate a series of malignancies, including colon cancer, metastatic breast cancer, and rhabdomyosarcoma [63,64,65]. The most therapeutic mechanism of inducing tumor death by vincristine is to break the polymerization of mitotic spindle microtubules and continuous arrests cell division during metaphase. Regarding the signs of vincristine-induced hepatotoxicity, increased levels of serum ALT, ALP, and AST are revealed in relation to the altered liver architecture. Moreover, hepatic content of MDA is enhanced, along with the significant decrease of hepatic SOD, GPx, reduced GSH (GSHr), GST, indicating that oxidative stress is established. Concomitantly, higher mRNA expressions of interleukin-12 (IL-12), interleukin-14 (IL-14), Bax, p53, and cleaved caspase-3 are simultaneously observed in hepatocytes, which stand for the induction of apoptosis. Reduced intracellular mRNA levels of Bcl-2 can be measured as well, suggesting that uptake of vincristine will invoke ROS-triggered apoptotic and inflammatory effects in liver tissues and cells [66].

Oxymatrine is a quinolizidine alkaloid isolated from the Chinese medicine *Sophora flavescens* and adopted to treat chronic viral hepatitis, plaque psoriasis, and arrhythmia [67,68,69]. Besides the findings of beneficial actions, it is proved to worsen liver damage. When treated with oxymatrine, cell viability will be reduced while the rate of apoptosis will be enhanced, as the intracellular markers for apoptotic pathway containing pro-caspase-3, -8, -9, and Bax are increasing, associated with the decrease of Bcl-2. In addition, expressions of endoplasmic reticulum (ER) stress indicators have been altered as well, including the activation of glucose regulated protein (GRP78), C/EBP homologous protein (CHOP), cleaved caspase-4, phospho-c-Jun N-terminal kinase (p-JNK), inositol-requiring enzyme 1 (IRE1), activating transcription factor 6 (ATF6) and pancreatic ER kinase (PERK). If there is interference with ER stress inhibition, intercellular levels of ROS, p-JNK, and cleaved caspase-3 will be dramatically dropped. As a consequence, hepatotoxicity of oxymatrine is linked with ER stress-induced ROS production [70].

Triptolide, triptonide, and wilforgine, three principal ingredients of *Tripterygium wilfordii*, elicit their pharmacodynamics in anti-inflammatory and anti-autoimmune diseases, especially rheumatoid arthritis [71]. At present, the clinical application of *Tripterygium wilfordii* is confined on account of its narrow therapeutic window and the high incidence of severe hepatotoxicity and nephrotoxicity [72]. Metabolic pathways for triptolide and triptonide are hydroxylation and cysteine conjugation, whereas wilforgine undergoes oxidative metabolism and hydrolysis. However, triptolide was demonstrated to be the major contributor to *Tripterygium wilfordii*-induced hepatotoxicity through hydroxylated by cytochrome P3A (CYP3A) [73], since CYP3A was specifically picked out as the main isoform responsible for triptolide hydroxylation and activates hepatic P450 which facilities the aggravation of liver toxicity [74,75]. Meanwhile, metabolic eliminations of GSH, as well as the reduction of NAD(P)H and quinine oxidoreductase 1 (NQO1), was verified in triptolide-treated mouse, indicating the dysfunction of the defense system to scavenge the oxidative species [76,77]. The nuclear factor erythroid 2-related factor 2 (Nrf2) is a regulator of cell resistance to pro-oxidant substances, and further mediates the antioxidant response components to control the physiological and pathophysiological outcomes of oxidant exposure. Triptolide-induced hepatotoxicity not only contains the blockade of cytoplasmic and nuclear Nrf2 activation, but inhibits the transition of hepatic influx and efflux transporters responsible for the interchanges of compound across cell membranes, such as P-gp, multidrug resistance-associated protein 2 (MRP2), multidrug resistance protein 4 (MRP4), bile salt export pump, and organic anion transporting polypeptide 2 (OATP2), at transcription level, which exerts more significant efficiency than that at functional level [76]. Meanwhile, blood biochemical levels of ALT and AST are elevated in combination with the failure in hematopoiesis, reproductive, renal, and cardiac systems through 0.2 mg/kg triptolide administration [78].

### 2.6. Herbal Extracts

*Polygonum multiflorum* (*Heshouwu* in Chinese) has been used in China for centuries, with anticancer, anti-inflammation, and reinforcing kidney function effects [79,80,81], However, accumulating evidence of liver cell damage arising from *Polygonum multiflorum* consumption has been reported, especially acute injury in morphological alteration in a time-dependent manner [82]. In 2013, the department of China Drug and Food Administration released a warning public announcement that more than 100 case reports concerning hepatotoxicity with *Polygonum multiflorum* treatment Investigation of Liver Injury of *Polygonum multiflorum Thunb*. Thus, a scientific basis for the toxicological mechanism of *Polygonum multiflorum* needs to be clarified. *Polygonum multiflorum* has two medicinal forms, *Polygoni multiflori radix* and *Polygoni multiflori radix prapaerata*. Notably, there is growing interest in the paradoxical effect of *Polygonum multiflorum* regarding whether it is hepatotoxic or not. Both forms share identical therapeutic effects in combating nonalcoholic fatty liver disease (NAFLD), fibrosis, as well as the cirrhosis, when the daily intake is less than 6 g per person [70,83,84]. The frequent mechanism of hepatotoxicity for both forms may contain cell cycle arrest and facilitate the activities of ALT, AST, ALP, creatinine, total bilirubin (TBil), direct bilirubin (DBil), and indirect bilirubin (IBil), and the leakage of LDH, whereas drug metabolic enzymes of cytochrome P3A4 (CYP3A4), cytochrome P2C19 (CYP2C19), CYP2E1, and SOD are attenuated by different pharmacokinetic behaviors [70,85,86,87]. The principal ingredients of *Polygonum multiflorum* include emodin-*O*-hex-sulfate, tetrahydroxystilbene-*O*-(galloyl)-hex. Among these, emodin and *cis*-stilbene glucoside might be the major responsibilities for liver toxicity [87]. In particular, *cis*-stilbene glucoside extracted from *Polygonum multiflorum* can trigger immunological idiosyncratic liver dysfunction in rats with lipopolysaccharide (LPS) intervention by repressing peroxisome proliferator-activated receptor (PPAR-γ) [88]. Aside from that, the ethanol extract is more toxic than the aqueous extract [89]. A method of prolonged decoction was convincingly demonstrated to effectively detoxicate *Polygonum multiflorum* [59]. Based on these alterations in biomarkers, *Polygonum multiflorum*-induced disturbance in the metabolic process of fat, bile acid, and amino acid may be the dominant threats to the induction of oxidative hepatotoxicity.

*Evodiae fructus*, an eminent Chinese herbal medicine, demonstrated its therapeutic capabilities in regard to anti-analgesic antiemetic and anti-inflammatory effects in gastrointestinal and cardiovascular diseases [90,91]. Alkaloids, including evodiamine and rutaecarpine, are the major bioactive ingredient in *Evodiae fructus*, and were reported to alleviate colorectal carcinoma, atherosclerosis and cardiovascular relaxation [92,93]. Nevertheless, the risk of increasing hepatotoxicity in patients treated with *Evodiae fructus* was frequently reported. Aqueous extract of *Evodiae fructus* can distinctly cause MPT in liver mitochondria, enhance the levels of AST, ALT, nitric oxide (NO), nitric oxide synthase (NOS), and MDA and downregulate the levels of MnSOD, GSH, and GPx, which synergistically lead to the occurrence of oxidative stress [94]. The cessation of ATP synthesis in association with induction of cytochrome C (CytC) release in hepatocytes was demonstrated in *Evodiae fructus*-dependent treatment, indicating the threshold of mitochondrial oxidative damage in liver [95,96].

*Genkwa flos* is characterized as the flower bud of *Daphne genkwa* and classified into the Chinese Pharmacopoeia with its wide range of pharmacodynamics actions including anti-herpes, anticancer, and inflammation-related symptoms [97,98,99]. Prior chemical studies have shown that *Genkwa flos* encompasses various types of constituents involving flavonoids, diterpenoids, lignans, and coumarins [100]. Current emerging evidence indicates that severe lesions to cardiac, renal, hepatic, and cutaneous tissues can be identified by excessive and prolonged administration of *Genkwa flos* [101,102,103,104]. Hepatotoxicity of *Genkwa flos* treatment have shown in HL-7702 liver cells, such as the increased hepatic serum makers of ALT, AST, and MDA, and downregulated oxidative stress indexes of CAT and GSH. In particular, phospholipase A2 (PLA2)/lysophosphatidylcholine (LPC) pathway is one of the crucial metabolic pathways involved in the glycerol phospholipid metabolism and participates in oxidative and inflammatory-induced liver injury [105]. It is remarkable that both the contents of PLA2 and LPC are significantly enhanced in *Genkwa flos*-treated HL-7702 liver cells [100]. The disruption of S1P metabolism mainly focused on Sphk/S1P pathway may occupy a vital position in *Genkwa flos*-related liver injury, as well [100]. Apart from that, chloroform extract of *Genkwa flos* demonstrated an inhibitory effect on the transcriptional activity of uridine diphosphate glucuronosyltransferase 1A1 (UGT1A1) and serum bilirubin, which can enhance the susceptibility to oxidative stress-induced chromosomal aberration during the liver injury [106].

*Cassia occidentalis*, mainly cultivated in the south of China and Asia, is adopted as a moderate purgative and stomachic herbal medicine. The ethanol extract of Cassia occidentalis has numerous therapeutic activities, including anti-inflammation and anti-anaphylaxis [107]. Several toxic reactions, including loss of weight, myopathy, and hepatocellular necrosis, have been noticed in animals fed with *Cassia occidentalis* [108,109]. In 2008, WHO reported that intermittent exposure to *Cassia occidentalis* can ultimately cause hepatomyoencephalopathy with failed muscle, hepatic, and cerebral dysfunction [110]. The transcriptional profiling demonstrated that gene expressions of antioxidant enzymes containing CAT and SOD have been reduced in *Cassia occidentalis*-treated rats. On the contrary, significant decreases in GPx and GSH expressions have been proofed, along with concomitant increase of lipid peroxidation, which re-emphasizes the involvement of oxidative stress [111,112]. Furthermore, profound reductions in xenobiotic-metabolizing enzymes, including CYP1A1, CYP1A2, CYP2B1, GST, and quinone reductase, have been validated in combination with the impairment of carbohydrate metabolism in *Cassia occidentalis*-treated rats, as well. Growing apoptotic and inflammatory factors, including Bax, caspase-3, NF-κB, TNF-α, Akt, TGF-β, MAPK-9 and -14, IL-6, JNK, p-38, and FasL, have been observed by microarray analysis in the hepatic tissue of rats fed with *Cassia occidentalis*, suggesting that multiple pathological pathways are involved and contribute to hepatocyte death [111]. Interestingly, acute administration of *Cassia occidentalis* alleviates oxidative stress in the kidney of *Rattus norvegicus*, which initially aims to treat hypertension by enhancing urinary excretion with the elimination of Na^+^, Cl^−^, and K^+^. Therefore, exact evidence for the underlying profile of *Cassia occidentalis* with hepatotoxicity deserves further studies [113].

## 3. Network Pharmacology-Associated Study

### 3.1. Network Construction and Targets Discovery

The general analogy of pharmacological actions is like a selective “key” that integrates into the specific “lock” of a drug target. The conception of creating selective ligands to counter undesirable adverse actions has been the dominant paradigm during the innovation of drug discovery. Nevertheless, post-genomic biology has revealed that the proposed schematic of drug action is far more complicated, suggesting that not only various “keys” may be suitable for a single “lock” but one “key” to fit multiple “locks”. Drug actions elaborated by means of network pharmacology can give insights into the pharmacological activities of hepatotoxic TCM, rather than separately enumerate case studies. As an innovative screening approach of drug target identification, network pharmacology prioritizes targets for TCM-induced oxidative hepatotoxicity in a systematic manner, aiming to identify the major pathological mechanism in hepatocytes.

**Figure 2:**
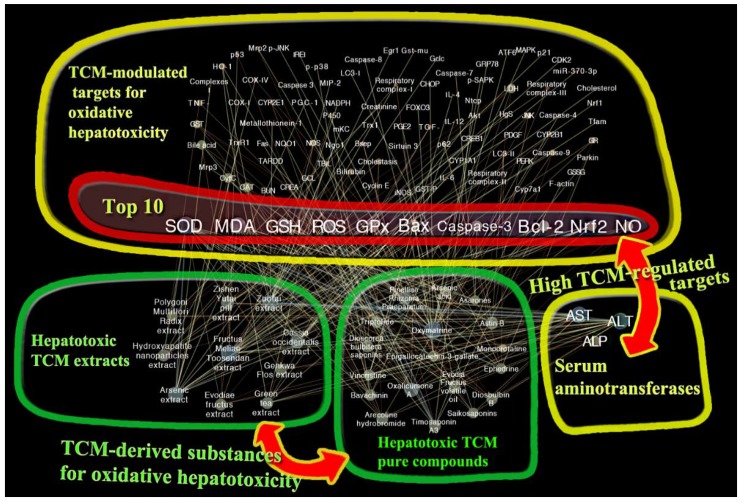
Network pharmacology-based target identification of TCM-derived natural compounds or extracts for the generation of oxidative hepatotoxicity.

**Figure 3:**
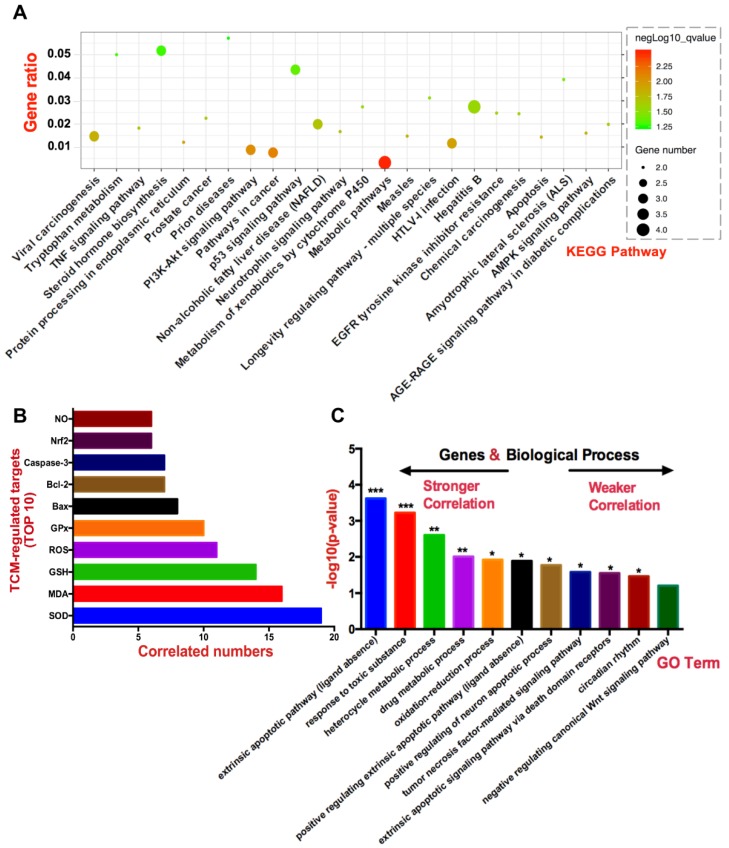
Target mining for TCM-caused oxidative hepatotoxicity by network pharmacology and bioinformatics enrichment analysis. (**A**) Scatter plot of enriched KEGG pathways statistics. The gene ratio illustrates the significantly expressed gene number to the total gene number in a certain pathway. (**B**) Identification of TCM-modulated targets for oxidative liver injury by network pharmacology. (**C**) GO analysis on the involvement of principal biological process. (*): −log10 (*p*) > 1.3; (**): −log10 (*p*) > 2, and (***): −log10 (*p*) > 3 versus background genes.

With regard to plotting the visualized network figure, the literature that meets the screening criteria mentioned in the section of “Literature Search Methodology” will be enrolled in the construction of a network. After a large-scale screening, the qualified data are imported in a bioscience software termed “Cytoscape” for network analysis (free access online at “http://www.cytoscape.org/”) [140,141]. Components of nodes in the network (Figure 2) refer to specific factors, including TCM-derived extracts, pure compounds, and TCM-modulated targets (protein or gene), whereas edges that straightforwardly interconnect nodes stand for TCM–target interactions. Those nodes with more shared edges and centripetal position account are more efficacious than those with less. For providing a deeper insight, network analysis has been performed by a statistical plug-in “NetworkAnalyzer” attached in Cytoscape, to calculate the correlation degree for each node with a specific parameter, along with typical size and color. More specifically, as shown in Figure 2, the larger and brighter the node is, the greater the degree of correlation is, which allows for the perception of differentiating the relativity of each node with oxidative hepatotoxicity through the color gradient from bright to dark (i.e., brightest-green; middle-yellow; darkest-red). Noticeably, since the serum aminotransferases of ALT, AST, and ALP are well-accepted indicators for reflecting the severity of liver diseases directly, the measurement of these three targets exists widely in the majority of liver disease studies, which results in the extremely high value of the degree of correlation [142]. Although we cannot postulate that oxidative hepatotoxicity is not associated with the alteration of ALT, AST, and ALP, these three serum aminotransferases with high correlation values will greatly interfere with the judgment of ranking other targets in relation to oxidative liver injury. Thus, during the statistical processing, these three factors will not be enrolled in the ranking of the TCM-regulated targets susceptible to oxidative stress-induced hepatotoxicity. Based on exquisitely programmed procedures by network pharmacology, the top 10 high influential factors modulated by TCM have been shown in Figure 3B, and are ranked in order as the following: SOD, MDA, GSH, ROS, GPx, Bax, caspase-3, Bcl-2, Nrf2, and NO, which play pivotal roles in the initiation and propagation of oxidative hepatotoxicity.

### 3.2. Hepatotoxic Role of Identified TCM-Regulated Targets by Network Pharmacology

#### 3.2.1. SOD, MDA, GSH, ROS, and GPx

Although oxidative hepatotoxicity caused by various medicines has been verified in numerous clinical trials, reports regarding the vital role of TCM-induced hepatic oxidative damage in patients are far away from satisfactory. In accordance with the principal targets picked up by NetworkAnalyzer, SOD is the most susceptible and regulated target for TCM-induced hepatic oxidative damage. When hepatocytes are attacked by TCM-derived compounds like atractyloside and saikosaponins, SOD will fail in preventing O_2_ overproduction when cell sensibility to hepatic injury is impaired by inhibiting the formation of peroxynitrite [96,143]. Serum levels of antioxidant enzymes, like SOD1, GPx, and CAT, are decreased in a patient with chronic liver cirrhosis and viral hepatitis when compared to healthy individuals. Inversely, indicators of oxidative stress and liver damage, such as MDA, NO, and ALT, are increased [144]. Upregulated ROS production in the formation of alcoholic liver disease (ALD) facilities the decreased of Cu-ZnSOD activity in association with the positively regulated NAD(P)H oxidase [145,146]. Interestingly, in certain special cases, even if the SOD content is steady or increased under pathological conditions, the possibility of defective SOD-induced oxidative damage may occur, since growing reports demonstrate that mutant forms of SOD may be generated, for example, in amyotrophic lateral sclerosis (ALS) disease. Mutations of SOD are proposed to modify the antioxidant proteins into pro-oxidants which are capable of invoking oxidative injury. However, whether this mechanism may exist in the development of hepatotoxicity or not, further studies are needed [144,147]. As for MDA, similar to 4-HNE, it is the end product of lipid peroxidation, representing a credible biomarker of oxidative stress. The formation of MDA can be extensively detected in ROS-caused degradation of polyunsaturated lipids [148]. Serum MDA levels usually increase in proportion to the severity of oxidative damage. Hereby, measuring MDA content may be considered as a reliable reference point for the degree of hepatic tissue impairment with oxidative stress. In addition, oxidative stress-related cirrhosis has been demonstrated to usually be linked with the negative regulation of MDA, SOD, GSH, and CAT [149]. ROS, undoubtedly acting as a dominant role in aerobic life, can be responsible for the manifestation of chronic liver disorders and stimulating their deterioration simultaneously. Referring to my previous description, ROS consists of various species, such as superoxide anion, hydrogen peroxide, and hydroxyl radicals. All of these substances possess specific inherent chemical characteristics and interact with different physiological and pathological targets. Hepatic cells, including Kupffer, endothelial, and stellate cells, are more susceptible to oxidative stress-induced apoptosis, necrosis, and tumorigenesis, especially by overproduced ROS [150].

#### 3.2.2. Bax, Caspase-3, and Bcl-2

Coincidentally, Bax, caspase-3, and Bcl-2 are all represented as notable biomarkers in cell apoptosis, particularly in mitochondrial apoptotic pathway [151]. It is noteworthy that mitochondrial permeability as well as transition potential can be disturbed by continuous ROS generation, phenomena which may not only result in the release of apoptotic inductors, such as Bax, caspase-3 and cytochrome C, but also the downregulation of Bcl-2, degradation of mitochondrial DNA, and dysregulation of ATP synthesis in an oxidative phosphorylation system [152,153,154]. In detail, Bcl-2 family members are evolutionarily conserved modulators for programmed cell death termed “apoptosis”, and both pro-apoptotic (i.e., Bax) and antiapoptotic (i.e., Bcl-2) members are affiliated in the same family. The ratio of Bax to Bcl-2 is broadly considered as a rheostat to measure the cell susceptibility to apoptosis. Caspase-3 is a cysteine protease that mediates apoptosis by proteolysis of particular substrates, especially by Bax/Bcl-2 [155]. Interestingly, in addition to being the downstream substrate of caspase-3, Bcl-2, with its antiapoptotic property, can be inactivated by caspase-3 and converted into a pro-apoptosis motivator unrelated to Bax/Bcl-2 pathway, suggesting that a feedback loop between Bcl-2 and caspase-3 exists [156]. Considerable evidence supports the idea that enhanced Bax/Bcl-2 ratio, combined with cleavage of caspase-3, takes place in various liver diseases, including chronic hepatitis, alcoholic liver, and hepatocellular carcinoma (HCC) [157,158,159].

#### 3.2.3. Nrf2 and NO

Nrf2, also known as nuclear factor (erythroid-derived 2)-like 2, is the principal modulator of encoding genes that protect cells against electrophilic stress. Thus, Nrf2 is comparatively less active in cells without stress, and coordinated with the basal flow of endogenic electrophile [160]. Activated Nrf2 in an oxidative environment not only illustrates the increasing synthesis of nucleophiles, including GSH and thioredoxin but, also, the enzyme-relevant catalysis for redox transitions [161]. As a sensor of cellular redox state, Nrf2 usually binds to Kelch-like epichlorohydrin-associated protein 1(KEAP1) in the normal physiological state. Increasing ROS, as well as electrophiles, can result in the release of Nrf2 into the nucleus, in order to activate the transcription of cell-protective genes. Antioxidant response element (ARE) can be activated to invoke antioxidant genes during oxidative stress [162]. However, ARE-associated genes, primarily regulated by Nrf2, govern GSH homeostasis and the activities of NAD(P)H quinone oxidoreductase 1 and uridine 5′-diphospho-glucosyltransferase [163]. Therefore, reduction of Nrf2 may make cells less sensitive to oxidative stress. Growing investigations have drawn attention to the alteration of Nrf2ARE feedback loop in counteracting the progression of various liver disorders including viral hepatitis, cirrhosis, hepatocellular carcinoma, and nonalcoholic liver disease [164,165,166,167]. Furthermore, this feedback loop associates with liver regeneration, as well. Overproduced ROS, derived from mitochondria, can be observed in Nrf2-knockout mice fed with pro-oxidant hepatotoxins or high-fat diet, along with more susceptibility to hepatotoxicity. Therefore, targeting Nrf2 as a curative target to treat oxidative stress-related liver diseases is meaningful and promising [168]. As for NO, it is a short-time lived free radical in gaseous state. It plays a variety of roles in the liver and other organs [168]. In healthy liver, NO production primarily originates from endotheliocytes by endothelial NO synthase (eNOS), and the low flow of NO is documented to adequately support the perfusion of liver sinusoids though regulating vascular tone and permeability [169]. Apart from that, endotheliocytes maintain sinusoid perfusion though upregulating NO generation. NO also regulates leukocyte adherence to sinusoidal endothelial cells, which is associated with attenuating the aggregation and adhesion of platelets [170]. In line with accelerating research studies, the expressions of NO and iNOS are positively regulated in almost all hepatocytes containing hepatic stellate cells under chronic hepatitis and endotoxemia [171,172]. In certain circumstances, NO has been deemed either a cytoprotective or a cytotoxic reagent, which depends on the local productive ratio of reactive oxygen intermediates (i.e., oxygen-centralized free radicals). Nevertheless, the predominant role of NO, in combination with the low contents of reactive oxygen intermediates is inclined towards the protective effect [173,174]. However, NO donors are reported to alleviate liver necrosis mainly by suppressing the levels of hydroxyl radical and lipid peroxidation, processes which can be blocked by the interaction between NO and reactive oxygen intermediates (i.e., lipid alkoxyl and lipid hydroperoxyl) and, in turn, attack the liver with overloaded NO [175,176]. In addition, upregulated serum aminotransferases of ALT, AST, and ALP may have strong linkage with oxidative hepatotoxicity, due to their high correlation with hepatic injury [177].

### 3.3. Bioinformatics Enrichment Analysis

Functional analysis of genes or signaling pathways in physiopathology are traditionally conducted among few clusters or even studied separately at a time. On the contrary, the lists of differential gene activities in most cases can be obtained and analyzed by gene-annotation databases with bioanalysis tools and experimental approaches, including DAVID (database for annotation, visualization and integrated discovery), KEGG (Kyoto encyclopedia of genes and genomes) database, KOBAS (KEGG orthology-based annotation system), microarray, and ChIP-on-CHIPs [178]. All these alternative technologies are indeed the bioinformatics scanning methods with sophisticated algorithms, rather than purely statistical tools. Gene-annotation enrichment study allows researchers to predict genome-wide genes under certain conditions and discern a series of specific biological processes most relevant to their investigation. Notably, the database of DAVID is equipped with powerful exploratory capacity for annotating, visualizing, and integrated discovering bioinformatics resources (available at https://david.ncifcrf.gov/home.jsp). Therefore, all the hepatotoxic TCM-modulated targets identified by network pharmacology have been incorporated into the integrated bioinformatics tool termed “Gene ontology (GO)” and “KEGG pathway analysis” in DAVID for identifying enriched biological pathways and highly correlated diseases (shared with similar participating genes) with kappa statistical analysis. Minus log (−log) transformed *p* value or *q* value (adjusted *p* value) have been input into GO and KEGG analysis, respectively (Figure 3A,C) followed by quantitatively estimating the statistical difference in comparison with background genes (background genes are usually automatically collected from backend database in DAVID). A value of −log10 (*p* or *q*) larger than 1.3 (equally in *p* < 0.05) is regarded as a significant difference. The figure of enriched KEGG pathways (Figure 3A) is plotted by R project (available at https://www.r-project.org)

Regarding this study, the methods of collecting biological information with a mode of “gene-to-annotation” is appropriate for mining primary TCM-modulated targets in triggering oxidative hepatotoxicity. After integrating network identified targets into the analytic tools of GO—Biological Process (BP) and KEGG in DAVID, KEGG result indicates that “metabolic pathway (−log10 (*q*) = 2.492)”, “pathways in cancers (−log10 (*q*) = 2.122)” and “P13K-Akt signaling pathway (−log10 (*q*) = 2.057)”, may have the most relevance to hepatotoxic TCM-modulated targets (Figure 3A). However, GO analysis in BP subunit illustrates that the most underlying pathways regulated hepatotoxic TCMs are “extrinsic apoptotic pathway in absence of ligand (−log10 (*q*) = 3.619)”, “response to toxic substance (−log10 (*q*) = 3.222)”, “heterocycle metabolic process (−log10 (*q*) = 2.602)”, and “drug metabolic process (−log10 (*q*) = 2.009)” (Figure 3C), indicating that intervention with metabolic process may play a vital role in TCM-dependent oxidative liver injury. Nevertheless, it is worth noting that management with targeting mining in a single database is limited with regard to providing comprehensive evidence. In this sense, heterogeneous databases with diverse enrichment tools should be taken into optimized analytic procedures in further study.

### 3.4. RUCAM (Roussel Uclaf Causality Assessment Method) in TCM-Induced Hepatotoxicity in Clinical Studies

#### 3.4.1. RUCAM-Based Causality Assessment

To cope with the increasing tendency of herb-induced liver injury (HILI), which includes herbal TCM-associated hepatotoxicity, clinicians have made great efforts to establish valid diagnostic criteria in the face of numerous clinical biomarkers [179]. In accordance with the recommendation in Asian and European regions, a causality assessment of HILI can be achieved by using a diagnostic tool RUCAM (Roussel Uclaf Causality Assessment Method), which provides a high degree of certainty [180,181]. Based on the systematical algorithms and quantitative method for identifying hepatotoxins and pharmacological hallmarks in case series, RUCAM is deemed as a validated mean of assigning key points for perceiving liver-specific clinical symptoms and cases. The resulting causality scores are marked in terms of core elements in updated RUCAM, such as time period and alterations of ALT values, etc. Of note, [182]. RUCAM scale typically ranges from −9 to +14, which can be hierarchically categorized as the following: ≥9 (highly probable); 6–8 (probable); 3–5 (possible); 1–2 (unlikely); ≤0 (excludes causality) [182,183]. 

#### 3.4.2. Identified Hepatotoxic TCM in Case Reports Using RUCAM

Although our knowledge of characteristics related to HILI has been substantially enriched within decades, little amounts of TCM-associated HILI have been identified, not to mention the causality between TCM consumption and hepatotoxicity in RUCAM-dependent clinical trials [184]. Herein, several updated RUCAM-based case reports containing TCM administration have been reviewed in this study. Melchart et al. performed a prospective and large-scale study concerning TCM-hepatotoxicity on the basis of RUCAM [185]. In the research, a total of 21,470 patients without liver diseases were treated with 11 independent herbal TCMs, such as *Bupleuri radix*, *Scutellaeiae radix*, and *Glycyrrhizae radix*, etc. Finally, 26 patients (0.12%) experienced high values of ALT (≥5 × upper limit of normal). However, RUCAM-related causality grades for TCM-treated patients were probable in 8/26 patients (score = 6–8), possible in 16/26 (score = 3–5), and excluded in 2/26 (score ≤ 0). Therefore, 24 patients (0.11%) might undergo TCM-induced hepatotoxicity and the most suspicious TCMs with hepatotoxic effects in this study were *Bupleuri radix* and *Scutellaeiae radix*, suggesting that these two herbs are mainly prone to induce liver injury with the causality grading of “possible”. Additional insights should be focused on the treatment of greater celandine and kava, since several RUCAM-based causality assessments indicated that hepatitis and liver cell necrosis could be generated in either greater celandine- or kava-treated patients. Both greater celandine and kava hepatotoxicity are primarily considered as an idiosyncratic liver injury in most susceptible individuals. More specifically, the causality gradings of 12 patients treated with greater celandine were probable or highly probable, while scores of highly probable, probable, or possible gradings could be marked in 8 patients with kava intervention, indicating that hepatic histological activity is more vulnerable to greater celandine or kava treatment [186,187,188,189]. Hao et al. has analyzed the etiology, clinical features, and prognosis of antipyretic analgesic drugs, antibiotics, and TCM-induced hepatotoxicity, including *Tripterygium wilfordii*, *Eucommia ulmoides*, and others. According to RUCAM-associated causality assessments in these HILI cases, TCM has emerged as a potential hepatotoxin (score > 3), with the grading at least “possible”, which is identical to both of the antipyretic analgesic drugs and antibiotics (both scores > 3) [190]. Zhang et al. have conducted literature mining targeting hepatotoxic TCM with RUCAM-based high causality grading. As a result, *Polygonum multiflorum* was documented to be the high probable TCM in 65 of 114 cases, a finding which was consistent with the experimental evidence discussed in this review [191].

### 3.5. Ethnopharmacology-Associated Challenges and Threats

Until now, accumulating evidence has especially manifested that liver injury may be the result of a therapeutic drug including TCM treatment. On the basis of both TCM theory and aforementioned findings in this study, principal strict challenges and threats can be summarized in the following seven aspects: (a) The criteria for distinguishing and identification of hepatotoxic TCM is ambiguous. Only a minority of hepatotoxic TCMs have been approved with safety issues by the department of China Drug and Food Administration. (b) Systematic screening and research studies focused on discovering and elaborating potential toxic targets for liver injury are greatly limited and time-dependent. Therefore, the findings gained from a certain period may have a deviation from the truth. (c) Most of the investigations concentrate on TCM-dependent pure compounds rather than TCM formula-caused liver dysfunction. Noticeably, TCM formula, which consists of multiple medicinal herbs, is the predominant form applied for treating diseases by TCM practitioners, rather than using a compound or refined extraction from a single herb. Therefore, growing attention should be drawn on disclosing the hepatotoxic effects involved in TCM formula, which seems to be equally meaningful. (d) A majority of studies regarding TCM-induced hepatotoxicity are still under the experimental stage and inadequate in multicenter clinical trials on a large scale. (e) Hepatotoxic role of TCM is composed of diverse pathological mechanisms and not limited to oxidative damage. Such hepatotoxicity-related pathogenesis as inflammation and apoptosis have crosstalk with oxidative stress. Hereby, it is uncertain which pathology plays a predominant and uncontestable role in hepatotoxicity development. (f) Due to the complexity of biological activities, interpretations of the association between numerous genes and relevant pathways should be performed with various advanced algorithms and annotation terms, aiming to avoid incomprehensive data mining. (g) Last but not least, assessment of risk-to-benefit ratio concerning the pharmacological actions of a novel anti-disease drug, including TCM or Western medicine, is imperative, and should be strictly conducted before any clinical applications.

## 4. Conclusions and Prospects

Regardless of the “holistic” and “natural” therapeutic modalities of TCM in response to various functional abnormalities, escalating interests are related to the cytotoxic role of TCM in the perturbation of organic physiological activities. Current investigations, including this study, have documented that oxidative stress usually participates in progressive hepatotoxicity, particularly triggered by TCM-regulated targets in hepatocytes (Figure 1). However, although liver is equipped with a well-established defense mechanism to protect hepatocytes from oxidative impairment, the intervention of potential toxic TCM remains to successfully invoke oxidative hepatotoxicity. Depending on the target mining by network pharmacology, 10 predominant factors, susceptible to hepatic oxidative injury upon TCM therapy, have been identified, including SOD, MDA, GSH, ROS, GPx, Bax, Caspase-3, Bcl-2, Nrf2, and NO. In addition, TCM-induced hepatotoxicity may be mainly involved in a metabolic pathway in accordance with bioinformatics enrichment analysis. Thus, practitioners should make an effort to be aware of the underlying hepatotoxic hazards prior to TCM application, but not be limited to focusing attention on the limited findings in this review. Selective TCM therapies to multiple diseases without ineluctable hepatotoxicity are essential and should be further studied.

## Figures and Tables

**Table 1:** Summary on the properties of TCM-induced hepatotoxicity in the recent 5 years.

Natural Compound	Sources of Chinese Medicine	Study Type	Cell or Animal	Biochemical Markers of Hepatotoxicity	Type of Injury	Reporting Date	Ref.
Vincristine	*Catharanthus roseus*	In vivo	Wistar rat	ALT, AST, IL-12. IL-4, p53, cleaved caspase-3, Bax↑; Bcl-2↓	Hepatitis	2018	[66]
Epigallocatechin-3-gallate	*Green tea*	In vivo	C57BL/6 mouse	SOD, GPx, respiratory complex-I -III, sirtuin 3, FOXO3, Nrf2↓	Hepatitis and hemorrhage	2018	[114]
Oxymatrine	*Sophora flavescens*	In vitro	L-02 cells	Pro-caspase-3 -4 -8 -9, GRP78, CHOP, p-JNK, IREI, ATF6, PERK, Bax, MDA, ROS↑; SOD, Bcl-2↓	Cell apoptosis	2018	[115]
Bavachinin	*Fructus psoraleae*	In vitro	HepaRG cells	JNK, p-p38, ROS, MAPK, MDA↑; SOD, GSH, CAT↓	Cell necrosis	2018	[116]
Genkwa Flos extract	*Genkwa flos*	In vitro & in vivo	HL-7702 cells; SD rat	ALT, AST, MDA↑; CAT, GSH, SOD, NO, NOS↓	Metabolism Dysregulation	2018	[117]
Fructus Meliae Toosendan extract	*Fructus meliae toosendan*	In vivo	BALB/c mouse	ALT, AST, MDA, p53, p21, Cyclin E, Bax, CytC, caspase-3 -9, CDK2, ROS↑; Bcl-2, Nrf2, miR-370-3p↓	Cell apoptosis	2018	[118]
Oxalicumone A	*Penicillium oxalicum*	In vitro	L-02 cells	ALT, AST, ROS, Caspase 3, MDA, NO, Fas, Bax, LDH, CytC↑; Bcl-2, GSH, SOD↓	Cell apoptosis	2018	[119]
Arsenic extract	*Arsenic*	In vitro	HHL-5 cell	Thioredoxin 1 (Trx1), TrxR1, ROS↑; Bax, CytC, Bcl-2↓	Cell apoptosis	2018	[120]
Pinelliae Rhizoma Praeparatum	*Pinelliae rhizoma*	In vivo	ICR mouse	ALT, AST, ALP, bile acid, Mrp3, MDA↑; SOD, GSH, GPx, Bsep, Mrp2, Nrf2↓	Metabolism dysregulation	2018	[121]
Hydroxyapatitenanoparticles extract	*Hydroxyapatite nanoparticles*	In vitro & In vivo	BRL cells; SD rat	TNF-α, NO, MDA, ROS↑; respiratory complex-I, -II, -III, GSH, SOD↓	Metabolism dysregulation	2018	[122]
Zishen Yutai pill extract	*Zishen yutai pill*	In vivo	SD rat	AST, ALP, ALT, MDA, LDH, PDGF, Cholestasis, Bile acid↑; SOD, GPx↓	Cell necrosis	2017	[123]
Polygoni Multiflori Radix extract	*Polygonum multiflorum thunb*	In vivo	SD rat	ALT, AST, ALP, LDH, bilirubin, creatinine↑SOD↓	Metabolism dysregulation	2017	[70] [124]
Arsenic acid	*Arsenic*	In vivo	Wistar rat	MDA, NO↑; SOD, GSH, GST, GPx↓	Metabolism dysregulation	2017	[125]
Saikosaponins	*Radix bupleuri*	In vitro & In vivo	HepG2 cells; Kunming mouse	CYP2E1, AST, ALT, LDH, ROS, iNOS↑; GSH↓	Metabolism dysregulation	2017	[62]
Ephedrine	*Ephedra sinica*	In vitro	LX-2 cells	Parkin, SOD2, ROS, Cox IV, p62, LC3 I, LC3 II↑	Excessive Mitophagy	2017	[126]
Arsenic extract	*Arsenic*	In vivo	Wister rat	Bax, caspase-3↑, CytC, SOD, complexes I, COX-I-IV, NRF-1-2, PGC-1α, Tfam↓	Metabolism dysregulation	2016	[127]
Dioscorea Bulbifera saponins	*Dioscorea bulbifera*	In vitro & In vivo	L-02 cells; Wister rat	ALT, AST, cytochromes P450, cholestasis↑; SOD, GPx, GST, GR, GCL↓	Metabolism dysregulation	2016	[43]
Zuotai extract	*Zuotai*	In vivo	Kunming mouse	ALT, AST, HgS, MeHg, metallothionein-1, heme oxygenase-1 (HO-1), Egr1, Gst-mu, mKC, MIP-2, NAD(P)H, Nqo1, Gclc↑	Cell inflammation	2016	[128]
Oxymatrine	*Sophora flavescens*	In vivo	ICR mouse	ALT, AST, ALP, TNF-α, caspase-9, -8, -3, TRADD, p-SAPK, p-JNK↑	Cell apoptosis	2016	[129]
Evodia Fructus volatile oil	*Evodia fructus*	In vivo	Kunming mouse	ALT, AST, PGE2, MDA, NO, NOS↑; SOD, GSH, GPx↓	Metabolism dysregulation	2015	[130]
Fructus Meliae Toosendan extract	*Fructus meliae* *toosendan*	In vivo	BALB/c mouse	ALT, AST, ALP, bilirubin, cholesterol↑; Nrf2↓	Cell necrosis	2015	[131]
Triptolide	*Tripterygium wilfordii*	In vivo	Kunmingmouse	ALT, AST, blood urea nitrogen (BUN), CREA↑; GSH↓	Acute hepatic necrosis	2015	[77]
Asarones	*Asarum*	In vitro	THLE-2 cells	Caspase-3 -7, MDA↑; GSH, GSSG↓	Cell apoptosis	2015	[132]
Timosaponin A3	*Anemarrhena asphodeloides*	In vivo	SD rat	Bile acid, ROS, HO-1↑; Ntcp, Bsep, Mrp2, Cyp7a1, F-actin↓	Metabolism dysregulation	2014	[133]
Astin B	*Aster tataricus*	In vitro	L-02 cells	ROS, JNK, CytC, Bax, caspases-9, -3, LC3-II↑; GSH, Bcl-2, p62↓	Cell apoptosis and inflammation	2014	[134]
Cassia Occidentalis extract	*Cassia occidentalis*	In vivo	Wister rat	TGF-β, JNK, Bax, MDA↑; Akt, CREB1, CYP1A1, CYP2B1, CAT, SOD1, IL-6, SOD, GR↓	Metabolism dysregulation and apoptosis	2014	[112]
Arecoline Hydrobromide	*Areca catechu*	In vivo	Wister rat	ALT, AST, MDA, CYP2B, CYP2E1↑; SOD, CAT, GPx, GSH↓	Liver cirrhosis and HCC	2014	[135]
Diosbulbin B	*Dioscorea bulbifera*	In vivo	ICR mouse	ALT, AST, ALP, MDA↑; GPx, GST, SOD, CAT↓	Metabolism dysregulation	2014	[136]
Evodiae Fructus extract	*Evodiae fructus*	In vivo	SD rat	MDA, CytC, AST, ALT, NO, NOS↑; SOD, GSH, GPx↓	Cell necrosis	2014	[94] [95]
Gardeniae Fructus extract	*Gardeniae fructus*	In vivo	SD rat	ALT, AST, ALP, bile acid, MDA, TNF-α, Bax↑; SOD, GPx, Bcl-2↓	Cell inflammation, necrosis and apoptosis	2014	[137]
Green tea extract	*Green tea*	In vivo	SD rat	ALT, AST, ALP, TBil, bilirubin, caspase-3, MDA, TG, GST-P↑	Metabolism dysregulation and apoptosis	2014	[138]
Monocrotaline	*Rattlebush*	In vivo	SD rat	GSH, GR, GPx, GST↓	Metabolism dysregulation	2014	[139]

In the Table 1, the symbols of “↑” and “↓” respectively represent for upregulated (“↑”) or downregulated (“↓”) biochemical markers by traditional Chinese medicine (TCM) treatment.

## Abbreviations

ALD	alcoholic liver disease
ALP	alkaline phosphatase
ALS	amyotrophic lateral sclerosis
ALT	alanine transaminase
AST	aspartate aminotransferase
ATF6	activating transcription factor 6
ATP	adenosine triphosphate
CAT	catalase
CHOP	C/EBP homologous protein
CytC	cytochrome C
CYP2C19	cytochrome P2C19
CYP2E1	cytochrome P2E1
CYP3A	cytochrome P3A
CYP3A4	cytochrome P3A4
CYP450	cytochrome P450
DAVID	database for annotation, visualization and integrated discovery
DBil	direct bilirubin
eNOS	endothelial NO synthase
ER	endoplasmic reticulum
GPx	glutathione peroxidase
GRP78	glucose regulated protein
GSH	glutathione
GST	glutathione *S*-transferase
HCC	hepatocellular carcinoma
HCV	hepatitis C virus
HILI	herb-induced liver injury
IBil	indirect bilirubin
IL-6	interleukin 6
IL-12	interleukin-12
IL-14	interleukin-14
IRE1	inositol-requiring enzyme 1
iNOS	inducible nitric oxide synthase
JNK	c-Jun N-terminal kinases
Keap1	Kelch-like ECH-associated protein-1
KEGG	Kyoto encyclopedia of genes and genomes
KOBAS	KEGG orthology-based annotation system
LDH	lactate dehydrogenase
LPC	lysophosphatidylcholine
LPS	lipopolysaccharide
MDA	malondialdehyde
NA(D)PH	nicotinamide adenine dinucleotide phosphate oxidase
NAFLD	non-alcoholic fatty liver disease
NO	nitric oxide
NOS	nitric oxide synthase
NQO1	NAD(P)H dehydrogenase, quinone 1
Nrf1	nuclear respiratory factor 1
Nrf2	nuclear factor (erythroid-derived 2)-like 2
MPT	mitochondrial permeability transition
MRP2	multidrug resistance-associated protein 2
MRP4	multidrug resistance protein 4
OATP2	organic anion transporting polypeptide 2
p38 MAPK	p38 mitogen-activated protein kinase
p-JNK	phospho-c-Jun N-terminal kinase
PERK	pancreatic ER kinase
PKC	protein kinase C
PLA2	phospholipase A2
PPAR-α	peroxisome proliferator activated receptor α
PPAR-γ	peroxisome proliferator-activated receptor-γ
RNS	reactive nitrogen species
ROS	reactive oxygen species
RUCAM	Roussel Uclaf Causality Assessment Method
SOD	superoxide dismutases
TBil	total bilirubin
TGF-β	transforming growth factor- β
TNF	tumor necrosis factor
UGT1A1	uridine diphosphate glucuronosyltransferase 1A1
4-HNE	4-hydroxynonenal
